# Development of novel O-polysaccharide based glycoconjugates for immunization against glanders

**DOI:** 10.3389/fcimb.2012.00148

**Published:** 2012-11-27

**Authors:** Mary N. Burtnick, Christian Heiss, A. Michele Schuler, Parastoo Azadi, Paul J. Brett

**Affiliations:** ^1^Department of Microbiology and Immunology, University of South AlabamaMobile, AL, USA; ^2^Complex Carbohydrate Research Center, The University of GeorgiaAthens, GA, USA; ^3^Department of Comparative Medicine, University of South AlabamaMobile, AL, USA

**Keywords:** *Burkholderia mallei*, *Burkholderia thailandensis*, O-polysaccharide, glycoconjugate, vaccine, immunization

## Abstract

*Burkholderia mallei* the etiologic agent of glanders, causes severe disease in humans and animals and is a potential agent of biological warfare and terrorism. Diagnosis and treatment of glanders can be challenging, and in the absence of chemotherapeutic intervention, acute human disease is invariably fatal. At present, there are no human or veterinary vaccines available for immunization against disease. One of the goals of our research, therefore, is to identify and characterize protective antigens expressed by *B. mallei* and use them to develop efficacious glanders vaccine candidates. Previous studies have demonstrated that the O-polysaccharide (OPS) expressed by *B. mallei* is both a virulence factor and a protective antigen. Recently, we demonstrated that *Burkholderia thailandensis*, a closely related but non-pathogenic species, can be genetically manipulated to express OPS antigens that are recognized by *B. mallei* OPS-specific monoclonal antibodies (mAbs). As a result, these antigens have become important components of the various OPS-based subunit vaccines that we are currently developing in our laboratory. In this study, we describe a method for isolating *B. mallei*-like OPS antigens from *B. thailandensis oacA* mutants. Utilizing these purified OPS antigens, we also describe a simple procedure for coupling the polysaccharides to protein carriers such as cationized bovine serum albumin, diphtheria toxin mutant CRM197 and cholera toxin B subunit. Additionally, we demonstrate that high titer IgG responses against purified *B. mallei* LPS can be generated by immunizing mice with the resulting constructs. Collectively, these approaches provide a rational starting point for the development of novel OPS-based glycoconjugates for immunization against glanders.

## Introduction

*Burkholderia mallei* is a non-motile, facultative intracellular, Gram-negative bacillus that causes a debilitating disease known as glanders. This zoonotic pathogen is an obligate animal parasite that is primarily responsible for disease in solipeds (i.e., horses, mules, and donkeys) (Howe and Miller, 1947; Redfearn et al., 1966; Yabuuchi et al., 1992; Srinivasan et al., 2001). Occasionally, in endemic regions, the organism may also cause disease in humans and other mammals (Miller et al., 1948). In equines, glanders presents as chronic or acute illnesses characterized by lung involvement, ulcerative nasal/tracheal lesions and visceral abscess formation. The clinical progression of human glanders is similar to that observed in solipeds and may manifest as chronic or acute localized infections, acute pulmonary infections or fulminating septicemias (Howe and Miller, 1947; Redfearn et al., 1966; Bartlett, 1998). Due to the potential use of *B. mallei* as an agent of biological warfare and terrorism, there is interest in developing effective glanders vaccines. To date, however, attempts to identify suitable candidates have been met with limited success.

Lipopolysaccharides, commonly referred to as endotoxins, are a major component of Gram-negative cell envelopes (Burns et al., 2006). The “barrier function” provided by bacterial outer membranes is largely due to the presence of these molecules (Nikaido, 2003). Bacterial strains expressing a “smooth” phenotype synthesize LPS antigens that are composed of three covalently linked domains: a lipid A moiety, a core region and an O-polysaccharide (OPS) (Raetz and Whitfield, 2002). Previous studies have shown that the OPS moieties expressed by *Burkholderia pseudomallei* (etiologic agent of melioidosis) and *Burkholderia thailandensis* (non-pathogenic saprophyte) are unbranched heteropolymers consisting of disaccharide repeats having the structure -3)-β-D-glucopyranose-(1-3)-6-deoxy-α-L-talopyranose-(1- in which ~33% of the 6-deoxy-α-L-talopyranose (L-6dTal*p*) residues possess 2-*O*-methyl and 4-*O*-acetyl substitutions while the remainder of the L-6dTal*p* residues bear only 2-*O*-acetyl modifications (Perry et al., 1995; Brett et al., 1998, 2003; Burtnick et al., 2002). Additionally, studies in our laboratory have also shown that *B. mallei* expresses OPS antigens that are structurally similar to those expressed by *B. pseudomallei* and *B. thailandensis* strains except that the L-6dTal*p* residues lack acetyl modifications at the *O*-4 position (Burtnick et al., 2002). This phenomenon certainly explains the ability of researchers to generate monoclonal antibodies (mAbs) specific for *B. pseudomallei* or *B. mallei* OPS antigens (Anuntagool and Sirisinha, 2002; Neubauer et al., 2005). Curiously, *B. mallei* isolates only appear to be capable of expressing a restricted repertoire of structurally diverse OPS antigens. It has even been suggested that virulent isolates of *B. mallei* can be defined by one serotype (Neubauer et al., 2005). At present, the significance of these observations with regards to virulence and evasion of host immune responses remain to be defined. Nonetheless, this phenomenon certainly bodes well from a vaccine development standpoint.

Virulent isolates of *B. mallei*, whether of human or veterinary origin, all appear to express smooth LPS phenotypes (Burtnick et al., 2002; Neubauer et al., 2005). Studies conducted in 1925 by Stanton and Fletcher demonstrated that *B. mallei* NCTC 120, now recognized as a rough isolate, was avirulent in both equine and rabbit models of infection (Stanton and Fletcher, 1925). More recently, we have shown that *B. mallei* strains, including NCTC 120, expressing rough LPS phenotypes are exquisitely sensitive to the bactericidal effects of normal human serum in comparison to those expressing a smooth phenotype thus implicating OPS as an important virulence determinant expressed by this pathogen (Burtnick et al., 2002). Additionally, and germane to the present study, Trevino et al. have shown that murine mAbs specific for *B. mallei* OPS are capable of passively immunizing mice against a lethal aerosol challenge (Trevino et al., 2006). Such findings confirm the protective capacity of this surface exposed antigen and support the rationale for developing OPS-based glycoconjugates for immunization against glanders.

In the present study, we describe the use of a variety of approaches to facilitate the development and preliminary testing of novel OPS-based glanders vaccine candidates. It is anticipated that via the application of these approaches, we will gain valuable insights toward the rational design of OPS-based glycoconjugates for immunization against disease caused by *B. mallei*.

## Materials and methods

### Strains and growth conditions

The bacterial strains used in this study are described in Table 1. Unless otherwise stated *B. pseudomallei*, *B. thailandensis* and *E. coli* were grown at 37°C on Luria Bertani-Lennox (LBL) agar or in LBL broth. For *B. mallei* and its derivatives, LBL media was supplemented with 4% glycerol (LB4G). When appropriate, antibiotics were added at the following concentrations: 25 μg/ml zeocin (Zeo) or 15 μg/ml polymyxin B (Pm) for *E. coli* and 5 μg/ml Zeo for *B. mallei*. Bacterial stocks were maintained at −80°C as 20% glycerol suspensions. All studies with *B. pseudomallei* and *B. mallei* were conducted in a CDC select agent certified biosafety level 3 containment facility.

**Table 1:** **Strains, plasmids, and primers**.

**Strains**	**Relevant characteristics**	**Source or reference**
***E. coli***		
TOP10	General cloning strain: Pm^s^, Zeo^s^	Invitrogen
S17-1	Mobilizing strain: Pm^s^, Zeo^s^	Simon et al., 1983
***B. pseudomallei***		
SZ210	DD503 derivative; Δ*wcbB*	Reckseidler-Zenteno et al., 2005
***B. mallei***		
ATCC 23344	Type strain; isolated in 1944 from a human case of glanders	Yabuuchi et al., 1992
GRS 23344	ATCC 23344 derivative; sucrose-resistant, Δ*sacB*: Pm^r^, Zeo^s^	Schell et al., 2008
BM210	GRS 23344 derivative; Δ*wcbB*: Pm^r^, Zeo^s^	This study
***B. thailandensis***		
DW503	ATCC 700338 (E264) derivative; Δ (*amrR-oprA*): Pm^r^, Zeo^s^	Burtnick et al., 2001
ZT0715	DW503::pZT0715: Pm^r^, Zeo^r^	Brett et al., 2011
***Plasmids***		
pEX18Zeo	Gene replacement vector; *oriT sacB*: Zeo^r^	Burtnick et al., 2010
pEXΔwcbB	pEX18Zeo harboring *wcbB* with an internal 792-bp deletion: Zeo^r^	This study
***Primers*^a^**		
wcbB-FB	5’-GATC*GGATCC*GCGCGCCACTGGCCCCCGACGTAG-3’	This study
wcbB-RXb	5’-GATC*TCTAGA*ACGATCTCTCGTGCGGGCGAGCC-3’	This study

a Restriction sites are italicized.

### Recombinant DNA techniques

The plasmids and oligonucleotide primers used in this study are described in Table 1. DNA manipulations were performed using standard methods. Restriction enzymes and T4 DNA Ligase (New England BioLabs) were used according to manufacturer's instructions. PCR was performed using an Expand High Fidelity PCR System (Roche Applied Science) or GoTaq DNA Polymerase (Promega); 1M Betaine (Sigma) was included in all PCR reactions. PCR was performed using the following conditions: 97°C for 5 min; 30 cycles, each consisting of 97°C for 45 s, 55°C for 45 s, and 72°C for 3 min; a final extension step of 72°C for 10 min was included. PCR and restriction digested products were purified using a QIAquick Gel Extraction Kit (Qiagen). Plasmids were purified using a QIAprep Spin Miniprep Kit (Qiagen). Genomic DNA was purified using a Wizard Genomic DNA Purification kit (Promega). Chemically competent *E. coli* TOP10 cells were transformed as per the manufacturer's instructions (Invitrogen). Oligonucleotide primers were obtained from Integrated DNA Technologies (Coralville, IA).

### Mutant construction

Gene replacement experiments with *B. mallei* were conducted using the *sacB*-based vector pEX18Zeo. To construct pEXΔwcbB, the wcbB-FB/wcbB-RXb primer pair was used to PCR amplify the Δ*wcbB* gene (792-bp markerless, in-frame deletion of the *wcbB* gene) from *B. pseudomallei* SZ210 genomic DNA (Reckseidler-Zenteno et al., 2005). The PCR product was then digested with BamHI and XbaI and cloned into pEX18Zeo digested with the same enzymes resulting in plasmid pEXΔwcbB.

To construct the *B. mallei* CPS mutant strain BM210, *E. coli* S17-1 was used to mobilize pEXΔwcbB into *B. mallei* GRS 23344 via conjugative mating essentially as previously described (Deshazer et al., 1997; Burtnick et al., 2011). Briefly, overnight cultures of S17-1 (pEXΔwcbB) and GRS 23344 were pelleted by centrifugation, resuspended together in 100 μ l of 10 mM MgSO_4_, spotted onto LB4G agar plates and incubated for 18 h at 37°C. To select for transconjugants, mating mixtures were plated onto LB-Zeo-Pm agar and incubated at 37°C for 48 h. To select for sucrose resistant colonies, individual transconjugants were inoculated into Yeast Tryptone (YT) broth, incubated at 37°C for 4–5 h, and then plated onto YT medium agar containing 5% sucrose (Hamad et al., 2009). Following incubation at 37°C for 48 h, sucrose resistant colonies were screened for loss of the Zeo resistance marker by replica plating onto LB4G and LB4G-Zeo. The resolved co-integrates were screened for the presence of the mutant allele (Δ*wcbB*) by PCR.

### LPS and OPS purification

LBL or LB4G broth (4 × 600 ml in 2 L baffled Erlenmeyer flasks) inoculated with the various *B. thailandensis* or *B. mallei* strains described in this study were incubated overnight at 37°C with vigorous shaking. Cell pellets were obtained by centrifugation (10 min at 8000× g) and extracted using a modified hot aqueous-phenol procedure (Perry et al., 1995). After extraction, the resulting phenol and aqueous phases were combined and dialyzed against distilled water to remove the phenol. The dialysates were then clarified by centrifugation (20 min at 10,000× g) and the supernatants concentrated by lyophilization. The crude LPS preparations were solubilized at 20 mg/ml in RD buffer (10 mM Tris-HCl [pH 7.5], 1 mM MgCl_2_, 1 mM CaCl_2_, 50 μg/ml RNase A and 50 μg/ml DNase I) and incubated for 3 h with gentle shaking at 37°C. Proteinase K was then added to a final concentration of 50 μg/ml and the samples were incubated for an additional 3 h at 60°C, whereupon the enzymatic digests were clarified by centrifugation (20 min at 10,000× g). LPS was then isolated from the supernatants as precipitated gels following successive rounds of ultracentrifugation (3 × 2 h at 100,000× g with the pellets being resuspended in ultrapure water between spins). After the final spin, the gel-like pellets were resuspended in ultrapure water and lyophilized.

To obtain purified OPS to synthesize the glycoconjugates, the *B. thailandensis* ZT0715 samples were solubilized at 5 mg/ml in 2% acetic acid (AcOH) and incubated for 2 h at 100°C. The hydrolyzed samples were cooled to room temperature, clarified via centrifugation (20 min at 10,000× g), and the supernatants were carefully removed and lyophilized. The lyophilized samples were then solubilized at 20 mg/ml in 100 mM phosphate buffered saline (pH 7.4; PBS) and clarified using 0.45 μm syringe filters. The samples were loaded onto Sephadex G-50 columns (40 × 2.6 cm) equilibrated with PBS and eluted with the same buffer. Fractions (6 ml) were collected and assayed for carbohydrate using the phenol-sulfuric acid method (Dubois et al., 1956). Carbohydrate positive fractions eluting near the column void volumes (~70 ml) were pooled, extensively dialyzed against distilled water and lyophilized. Protein and nucleic acid contamination of the resulting OPS preparations were estimated by BCA assay (Pierce) and A_260/280_ measurements, respectively.

### Glycosyl composition analysis

Column purified OPS (200 μ g) was mixed with 300 μ l 1 M methanolic HCl and incubated at 80°C in a sealed glass tube. After 16 h, the mixture was dried down under a stream of dry nitrogen and co-evaporated three times with dry MeOH to remove residual acid. After re-N-acetylation with acetic anhydride (100 μ l) in methanol/pyridine (2:1, 300 μ l), the sample was treated with 200 μ l Tri-Sil® (Pierce), incubated in a sealed tube for 20 min at 80°C, and dried down. The residue was taken up in 2 ml hexane, filtered, and dried down. The resulting TMS-methyl glycosides were dissolved in 150 μ l hexane and analyzed on an Agilent 7890A gas chromatograph interfaced to a 5975C mass selective detector in electron impact ionization mode. Separation was performed on a 0.25 μm EC-1 bonded phase fused silica capillary column (30 m × 0.25 mm).

### NMR spectroscopy

Column purified OPS samples were deuterium exchanged by dissolving in D_2_O, followed by lyophilization. The samples were then dissolved in D_2_O containing a trace amount of acetone, and ^1^H and ^13^C NMR spectra were obtained using a Varian Inova-500 MHz spectrometer at 50°C using standard pulse sequences. ^1^H and ^13^C chemical shifts were measured relative to the internal acetone reference (δ_H_ = 2.218 ppm; δ_C_ = 33.0 ppm) (Wishart et al., 1995).

### Glycoconjugate synthesis

Glycoconjugates were synthesized using established methodologies (Jennings and Lugowski, 1981; Brett and Woods, 1996; Conlan et al., 2002). Briefly, purified OPS samples were solubilized at 5 mg/ml in PBS and added to a small amber vials. To each ml of the solutions was added 6 mg (~30 mM) of sodium *meta*-periodate (NaIO_4_; Pierce). Once the crystals had dissolved by gentle agitation, the reaction mixtures were gently stirred at room temperature for 40 min. To remove any excess oxidizing agent, the reaction mixtures were applied to a Zeba Desalt Spin Columns (Pierce) equilibrated with PBS and the eluates collected. To facilitate conjugation of the OPS antigens to cationized bovine serum albumin (cBSA; Pierce), diphtheria toxin mutant CRM197 (CRM197; List Biological Laboratories, Inc.) or cholera toxin B subunit (CtxB; Sigma), the activated OPS samples were added to small amber vials. To each ml of the OPS solutions was added 0.5, 1, 2, or 4 ml of the carrier proteins (5 mg/ml in PBS). After mixing by gentle agitation, 10 μ l aliquots of a sodium cyanoborohydride stock (1 M NaBH_3_CN in 10 mM NaOH) were added to each ml of the conjugation mixtures and the reactions were gently stirred at room temperature for 4 d. Following this, 10 μ l aliquots of a sodium borohydride stock (1 M NaBH_4_ in 10 mM NaOH) were added to each ml of the conjugation mixtures and the reactions were stirred for 40 min. The conjugate reactions were then brought to 5 ml with ultrapure water, dialyzed against distilled water and then lyophilized. The resulting preparations were resuspended in ultrapure water as 1 mg/ml stocks and stored at -20°C until required for use. BCA assays were used to quantitate the protein concentrations of the glycoconjugate stocks.

### Antibody production

6–8 weeks old female BALB/c mice were immunized subcutaneously on days 0, 21, and 35 with 10 μ g of the OPS1B1 glycoconjugate (~5 μ g of OPS as a conjugate) formulated with saline, saline plus Alhydrogel 2% (500 μ g/mouse; Brenntag) or saline plus Alhydrogel 2% plus ODN 2006 (CpG; 20 μ g/mouse; InvivoGen). Unconjugated OPS and cBSA antigens (similarly formulated) served as controls. Terminal bleeds were conducted 7 days after the third immunization. All procedures involving the mice were performed according to protocols approved by the University of South Alabama Institutional Animal Care and Use Committee.

### SDS-PAGE and western immunoblotting

The LPS and glycoconjugate samples were solubilized in 1X SDS-PAGE sample buffer and heated to 100°C for 5 min prior to use. Proteins were separated by electrophoresis on Novex 12% Tris-Glycine gels (Life Technologies) and visualized via staining with Coomassie Blue R-250. For immunoblot analyses, the LPS and glycoconjugate samples were separated on the same 12% gels and electrophoretically transferred to nitrocellulose membranes. The membranes were blocked with 3% skim milk in high salt Tris-buffered saline (HS-TBS; 20 mM Tris, 500 mM NaCl, pH 7.5) for 60 min at room temperature and then incubated overnight at 4°C using 1/1000 dilutions of the *B. mallei* OPS-specific mAbs 3D11 (Research Diagnostics, Inc.) and 9C1-2 (Trevino et al., 2006) or 1/800 dilutions of the *B. pseudomallei*/*B. thailandensis* OPS-specific mAb Pp-PS-W (Bryan et al., 1994). To facilitate detection, the membranes were incubated for 1 h at room temperature using 1/5000 dilutions of anti-mouse IgG (for 3D11) or IgM (for Pp-PS-W) horse radish peroxidase conjugates (SouthernBiotech). The blots were then visualized using HRP Color Development Reagent (Bio-Rad) or Pierce ECL Western Blotting Substrate (Pierce).

### Quantitation of immunoglobulin titers by ELISA

Ninety-six well Maxisorp plates (Nunc) were coated overnight at 4°C with purified *B. mallei* BM210 LPS (10 μg/ml) solubilized in carbonate buffer (pH 9.6). The plates were blocked at room temperature for 30 min with StartingBlock T20 (TBS) Blocking Buffer (Pierce) and then incubated for 2 h at 37°C with the mouse serum samples serially diluted in Tris-buffered saline + 0.05% Tween 20 (TBS-T; pH 7.5) + 10% StartingBlock T20. To facilitate detection, the plates were incubated for 1 h at 37°C with 1/2000 dilutions of anti-mouse IgG, IgG_1_, or IgG_2_*_a_* horse radish peroxidase conjugates (SouthernBiotech). The plates were then developed with TMB substrate (KPL) and read at 620 nm. The reciprocals of the highest dilutions exhibiting ODs of >0.150 were used to determine the endpoint titers for the individual mice. The data was plotted and analyzed using GraphPad Prism 5 (GraphPad Software Inc.). Statistical differences between geometric mean IgG titers ware assessed by Mann–Whitney rank sum analysis with the significance set at *P* < 0.05.

### Cell culture and opsonophagocytosis assays

The murine macrophage cell line RAW 264.7 (ATCC TIB-71) was maintained in Dulbecco's modified Eagle's medium supplemented with 10% (v/v) heat inactivated (HI) fetal bovine serum (DMEM-10; Invitrogen) and a standard mixture of antibiotics (100 U ml^−1^ penicillin, 100 μ g ml^−1^ streptomycin and 250 μ g ml^−1^ amphotericin B; Sigma) at 37°C under an atmosphere of 5% CO_2_. Opsonophagocytosis assays were based upon previously described methods (Simon et al., 2011; Tennant et al., 2011). Briefly, RAW 264.7 cells resuspended in DMEM-10 were transferred into 24-well tissue culture plates at a density of ~1 × 10^6^ cells/well and incubated overnight. *B. mallei* ATCC 23344 cultures grown to early-log phase were pelleted, resuspended at a density of ~10^6^ cfu/ml in DMEM containing 0.5% vehicle/adjuvant only control or OPS1B1 mouse immune serum (pooled and HI for 30 min at 56°C) and then incubated at 37°C for 1 h. RAW 264.7 monolayers were washed twice with Hanks' Balanced Salts Solution (HBSS; Invitrogen) prior to the addition of the opsonized bacterial suspensions. The monolayers were incubated with the bacteria at 37°C under an atmosphere of 5% CO_2_ for 1 h and then washed twice with HBSS to remove extracellular bacteria. Infected RAW 264.7 cells were incubated in fresh DMEM-10 containing 250 μg/ml kanamycin to suppress the growth of residual extracellular bacteria. At 3 h post-infection, monolayers were washed twice with HBSS, lysed with 0.2% (v/v) Triton X-100 (Sigma) and serial dilutions of the lysates were plated onto LB4G agar and incubated at 37°C for 48 h. Plate counts were used to enumerate bacterial loads. The data was plotted and analyzed using GraphPad Prism 5. Statistical differences were assessed by *t*-test with the significance set at *P* < 0.05.

## Results and discussion

### Glycoconjugate vaccines

Immunologically, antigens can be classified as either T cell-dependent (TD) or T cell-independent Type 1 or Type 2 (TI-1 or TI-2) (Mosier et al., 1977a,b). In general, proteins and peptides are TD antigens while carbohydrates are TI-1 and TI-2 antigens (Weintraub, 2003). Polysaccharides such as capsular antigens and OPS are considered TI-2 antigens (Jones, 2005). Without the involvement of T cells, TI-2 antigens do not induce immunological memory, avidity maturation or isotype switching (Weintraub, 2003). Additionally, repeat vaccination does not evoke high antibody titer responses and without immunization at frequent intervals, antibody levels will decline (Jones, 2005). Typically, only high molecular weight TI-2 antigens are immunogenic due to their ability to crosslink multiple surface immunoglobulin (sIg) molecules present on individual B cells (Mond et al., 1995; Snapper and Mond, 1996). For this reason OPS and low molecular weight capsular polysaccharides alone are often not effective vaccine candidates.

The mechanism by which glycoconjugates stimulate immune responses involves initial recognition by sIg molecules displayed by antigen specific B cells. Following recognition of the carbohydrate component, glycoconjugates are internalized and the carrier protein is degraded by proteolytic enzymes. Peptides are then transported to and displayed by major histocompatibility complex (MHC) class II molecules. The peptide-loaded MHC class II molecules are then recognized by T helper (Th) cells, which provide appropriate signals through direct B cell/Th cell interactions and via cytokine signaling processes to induce maturation of the B cells into antibody secreting plasma and memory cells. Since cross-linking of multiple sIg molecules is not required during this process, glycoconjugate vaccines can be produced from low molecular weight carbohydrates such as OPS antigens (Kuberan and Linhardt, 2000; Lesinski and Westerink, 2001; Lockhart, 2003; Weintraub, 2003; Jones, 2005).

### Purification and characterization of *B. mallei*-like OPS antigens

OPS-based glycoconjugates represent a rational approach for immunizing against disease caused by *B. mallei*. To date, however, isolating OPS antigens for this purpose has been hampered by the fact that *B. mallei* is a select agent requiring specialized handling and containment practices. To address these issues, we have begun to investigate the use of a closely related but non-pathogenic *Burkholderia* species as a source of *B. mallei*-like OPS antigens. By doing so, we believe that it is possible to safely and cost-effectively produce these vaccine candidates for testing without the requirement for BSL-3 containment. Previous studies have shown that the OPS expressed by *B. mallei* are similar to those produced by *B. thailandensis* except that they lack the 4-*O*-acetyl modifications on their 6-deoxy-α-L-talopyranosyl residues (Burtnick et al., 2002). Recently, we described the identification and characterization of an open reading frame, designated *oacA*, expressed by *B. thailandensis* that accounts for this phenomenon. Utilizing the *B. thailandensis* and *B. mallei* OPS-specific mAbs, Pp-PS-W and 3D11 respectively, Western immunoblot analyses demonstrated that the OPS antigens expressed by the *oacA* mutant, *B. thailandensis* ZT0715, were antigenically similar to those produced by *B. mallei* ATCC 23344 (Brett et al., 2011).

**Figure 1:**
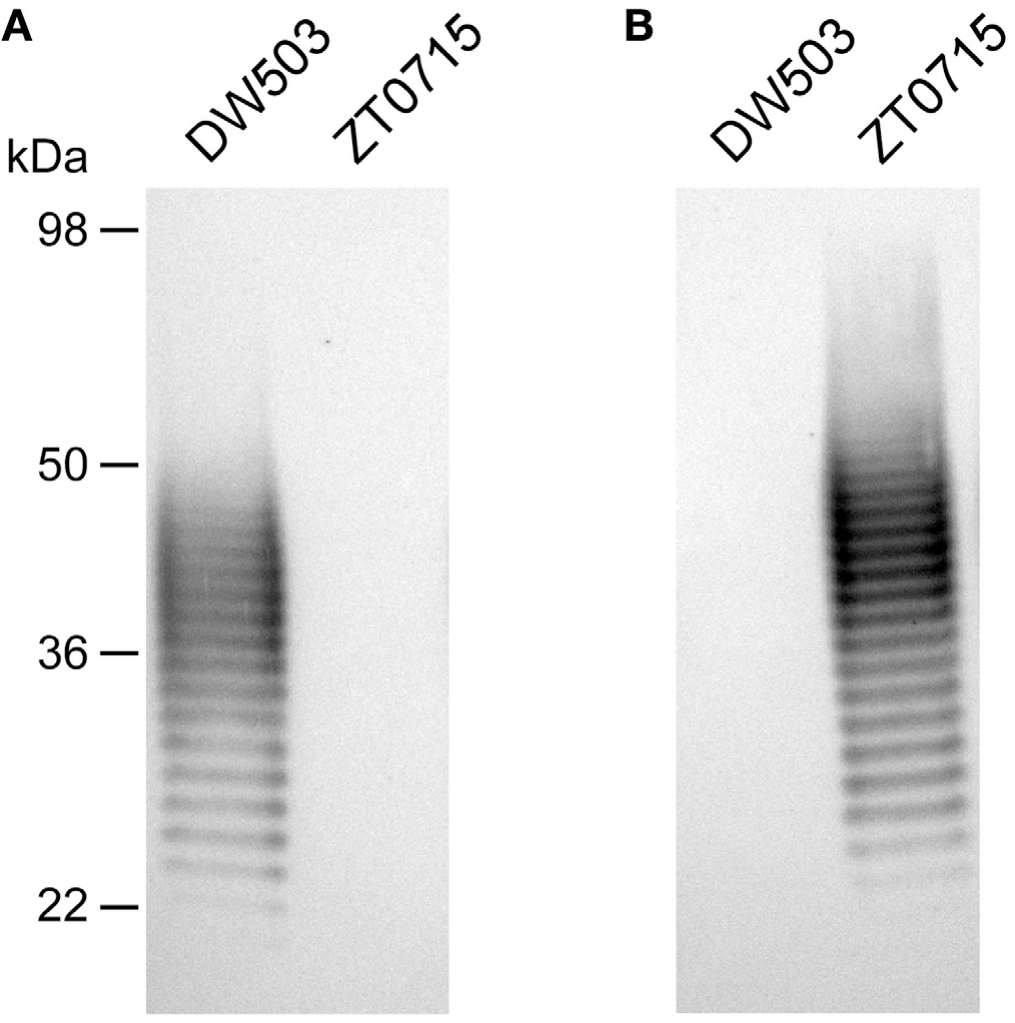
**Western immunoblot analysis of *B. thailandensis* LPS antigens.** Purified *B. thailandensis* DW503 (WT) and ZT0715 (*oacA* mutant) LPS antigens were probed with **(A)** the *B. thailandensis* OPS-specific mAb, Pp-PS-W or **(B)** the *B. mallei* OPS-specific mAb, 3D11. The positions of protein molecular size standards are indicated on the left.

To facilitate the construction of the glycoconjugates described in this study, LPS-antigens were isolated from *B. thailandensis* using a modified enzyme hot aqueous-phenol procedure (Brett et al., 2003, 2011). Consistent with previous observations, only the antigens purified from ZT0715 (*oacA* mutant) and not the parent strain (DW503) were shown to react strongly by Western immunoblotting with the *B. mallei* OPS-specific mAbs, 3D11 and 9C1-2 (Figure 1 and data not shown). We routinely obtain 20–25 mg of OPS per liter of ZT0715 culture by column purification of acid hydrolyzed LPS, with the resulting antigen preparations being devoid of detectable protein or nucleic acid contamination as determined by BCA assay or UV spectroscopy. To assess the structural integrity and homogeneity of the OPS preparations, samples are also subjected to glycosyl composition and NMR spectroscopy analyses. Using these approaches, we are able to demonstrate that the preparations consist primarily of the -3)-β-D-glucopyranose-(1-3)-6-deoxy-α-L-talopyranose-(1-heteropolymer in which the L-6dTal*p* residues bear only 2-*O*-acetyl modifications confirming their similarity to *B. mallei* OPS preparations (data not shown).

### Conjugation of OPS to carrier proteins

**Figure 2:**
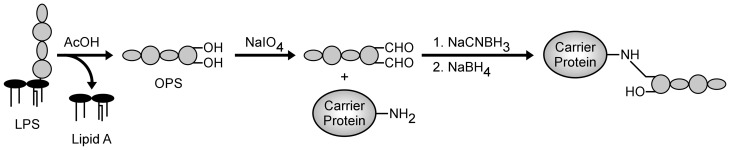
**Generalized scheme for the conjugation of chemically activated *B. thailandensis* OPS to carrier proteins**.

To facilitate the coupling of *B. thailandensis* OPS to protein carriers, we have found that NaIO_4_ is well suited for chemically activating the polysaccharide. The decision to use NaIO_4_ was based upon several considerations including (1) the absence of amines or carboxylates in the antigen that would favor conjugation via other methods, (2) the relative safety of the compound over alternatives such as cyanogen bromide, (3) the use of reaction conditions to minimize the risk of reducing alkali-sensitive *O*-acetyl modifications, (4) the desire to construct neoglycoconjugates rather than cross-linked network conjugates (Jones, 2005). Using the approach outlined in Figure 2, reactive aldehydes incorporated into the core residues at the reducing termini of *B. thailandensis* OPS facilitate the conjugation of the antigen to carrier proteins via reductive amination. To reduce the Schiff bases formed during the coupling reactions, NaBH_3_CN was chosen as the primary reducing reagent because, unlike NaBH_4,_ it rapidly modifies Schiff bases but not aldehydes. Once the OPS moieties are coupled to carrier proteins, however, NaBH_4_ is then added to quench any residual aldehydes in the reaction mixtures.

**Figure 3:**
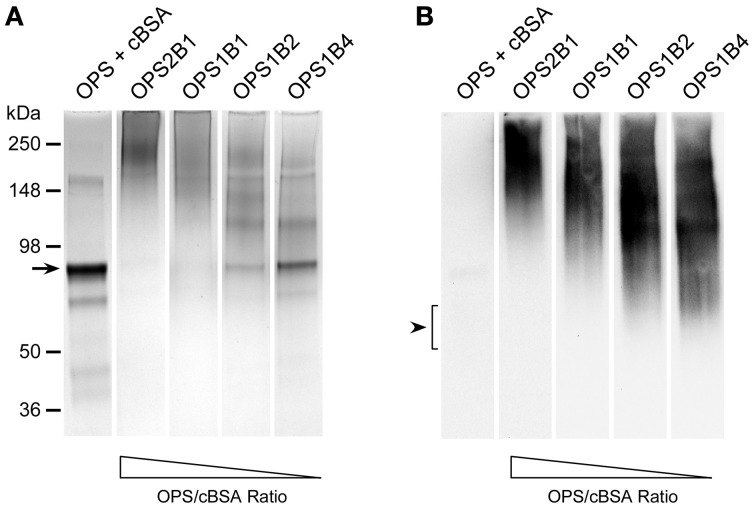
**Physical analysis of *B. thailandensis* OPS-cBSA glycoconjugates. (A)** SDS-PAGE and Coomassie Blue staining was used to confirm the covalent linkage of ZT0715 OPS to cBSA. The OPS + cBSA lane represents unconjugated controls. The black arrow indicates the position of the predominant cBSA band. The positions of protein molecular size standards are indicated on the left. **(B)** Western immunoblotting was also used to assess the antigenicity of the chemically activated/coupled ZT0715 OPS. OPS was detected using the 3D11 mAb. Lanes were loaded with similar amounts of protein or carbohydrate to facilitate direct comparisons. The black arrowhead indicates the position of the OPS which binds poorly to the membrane when unconjugated.

Initially, the OPS-based glycoconjugates synthesized in this study were constructed using cBSA as the protein carrier. The decision to use cBSA for this purpose was based on several considerations including the fact that it is (i) a well characterized immunogen, (ii) commercially available, and (iii) affordable. To facilitate our studies, glycoconjugates were synthesized by reacting various ratios of chemically activated OPS and cBSA with one another (e.g., 2:1, 1:1, 1:2, and 1:4 ratios of OPS:cBSA). Upon conjugation of the antigens, the samples were examined by SDS-PAGE. Results of these analyses indicated that in all instances, the OPS had covalently linked to the protein carrier as demonstrated by the shift in molecular weight of the cBSA relative to the unconjugated control (Figure 3A). In addition, Western immunoblotting confirmed that the structural integrity/antigenicity of the OPS moieties remained intact after chemical activation and linkage to the protein carrier based upon their reactivity with the mAbs, 3D11 and 9C1-2 (Figure 3B and data not shown). Furthermore, studies demonstrated that by varying initial carbohydrate to protein ratios, we could influence the molecular weights of the resulting glycoconjugates. It should be noted, however, that the glycoconjugates began to precipitate out of solution at the OPS:cBSA ratio of 2:1.

### Immune responses against OPS-based glycoconjugates

**Figure 4:**
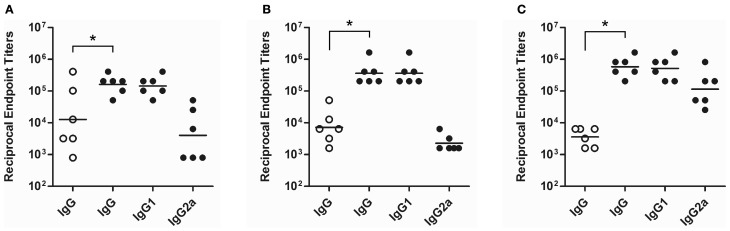
**Characterization of serum immunoglobulin responses following immunization of mice with the OPS1B1 glycoconjugate.** ELISA was used to determine the serum IgG responses of mice immunized with OPS1B1 (filled circles) or the unconjugated controls (OPS + cBSA; open circles). The OPS1B1 and control samples were formulated with **(A)** saline, **(B)** saline plus Alhydrogel or **(C)** saline plus Alhydrogel/CpG prior to immunization. Individual symbols represent single mouse while the black horizontal bars indicate geometric means. ^*^P < 0.05.

**Figure 5:**
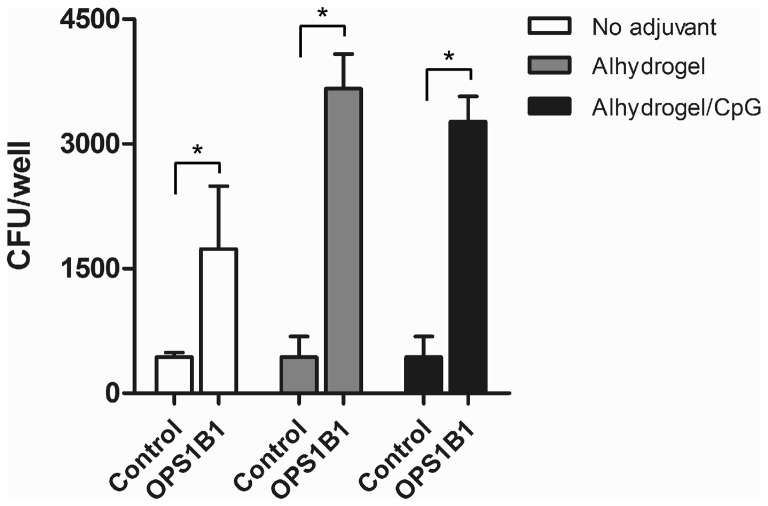
**Opsonophagocytic uptake of *B. mallei* by RAW 264.7 murine macrophages.** Bacterial uptake in the presence of HI pooled vehicle/adjuvant only control serum (Control; *n* = 6) or pooled anti-OPS1B1 immune serum (OPS1B1; *n* = 6) was quantitated at 3 h post-infection. Values represent the means ± SD of three independent experiments. ^*^P < 0.05.

Studies suggest that vaccine candidates promoting T helper 1 (Th1)-like cellular and humoral immune responses will likely be required to immunize against glanders (Amemiya et al., 2002, 2006). To examine the immunogenic potential of the cBSA-based glycoconjugates synthesized in this study, mice were immunized with either the OPS1B1 construct (49.3% protein on a w/w basis) or unconjugated controls. The primary reason for utilizing OPS1B1 was that of the four conjugates synthesized in this study, OPS1B1 was the one that possessed the highest percentage of OPS (on a w/w basis) while still remaining soluble. Analysis of serum samples obtained from the terminal bleeds demonstrated that, in all instances, mice immunized with OPS1B1 produced significantly higher anti-*B. mallei* LPS IgG titers than those immunized with the unconjugated controls (Figures 4A–C). Although the mice that were immunized with OPS1B1 in saline exhibited impressive anti-LPS IgG levels, as expected those that were immunized with the conjugate formulated with Alhydrogel or Alhydrogel/CpG produced the highest IgG titers (Table 2). Additionally, based upon the analysis of the IgG2a:IgG1 ratios, it appears that mice immunized with OPS1B1 formulated with saline or Alhydrogel produce T helper 2 (Th2)-like biased humoral responses while those immunized with the conjugate formulated with Alhydrogel/CpG produce mixed Th1/Th2-like humoral responses (Figures 4A–C and Table 2). To assess the functional activity of the OPS1B1 antiserum, samples were examined for their ability to mediate opsonophagocytic uptake. As shown in Figure 5, pre-incubation of *B. mallei* with HI pooled anti-OPS1B1 serum, but not HI pooled vehicle/adjuvant only control serum, significantly enhanced bacterial uptake into RAW 264.7 murine macrophages.

**Table 2:** **Effect of adjuvants on serum immunoglobulin responses**.

**Immunogen(s)**	**Adjuvant**	**Antibody titers^a^–Terminal bleed (1 SD confidence interval)**			
		**IgG**	**IgG1**	**IgG2a**	**Ratio IgG2a:IgG1**
OPS + cBSA	None	12,800 (1.07e3–1.53e5)	ND^b^	ND	−
OPS1B1	None	162,550 (7.67e4–3.45e5)	144,815 (6.75e4–3.11e5)	4032 (5.52e2–2.94e4)	0.028
OPS + cBSA	Alhydrogel	7,184 (2.05e3–2.52e4)	ND	ND	−
OPS1B1	Alhydrogel	364,912 (1.56e5–8.54e5)	364,912 (1.56e5–8.54e5)	2263 (1.23e3–4.16e3)	0.006
OPS + cBSA	Alhydrogel/CpG	3,592 (1.76e3–7.35e3)	ND	ND	−
OPS1B1	Alhydrogel/CpG	579,262 (2.70e5–1.24e6)	516,064 (2.14e5–1.25e6)	114,940 (3.03e4–4.37e5)	0.223

a Titers are reported as geometric means (*n* = 6 mice per group).

b ND, not determined.

## Conclusions

**Figure 6:**
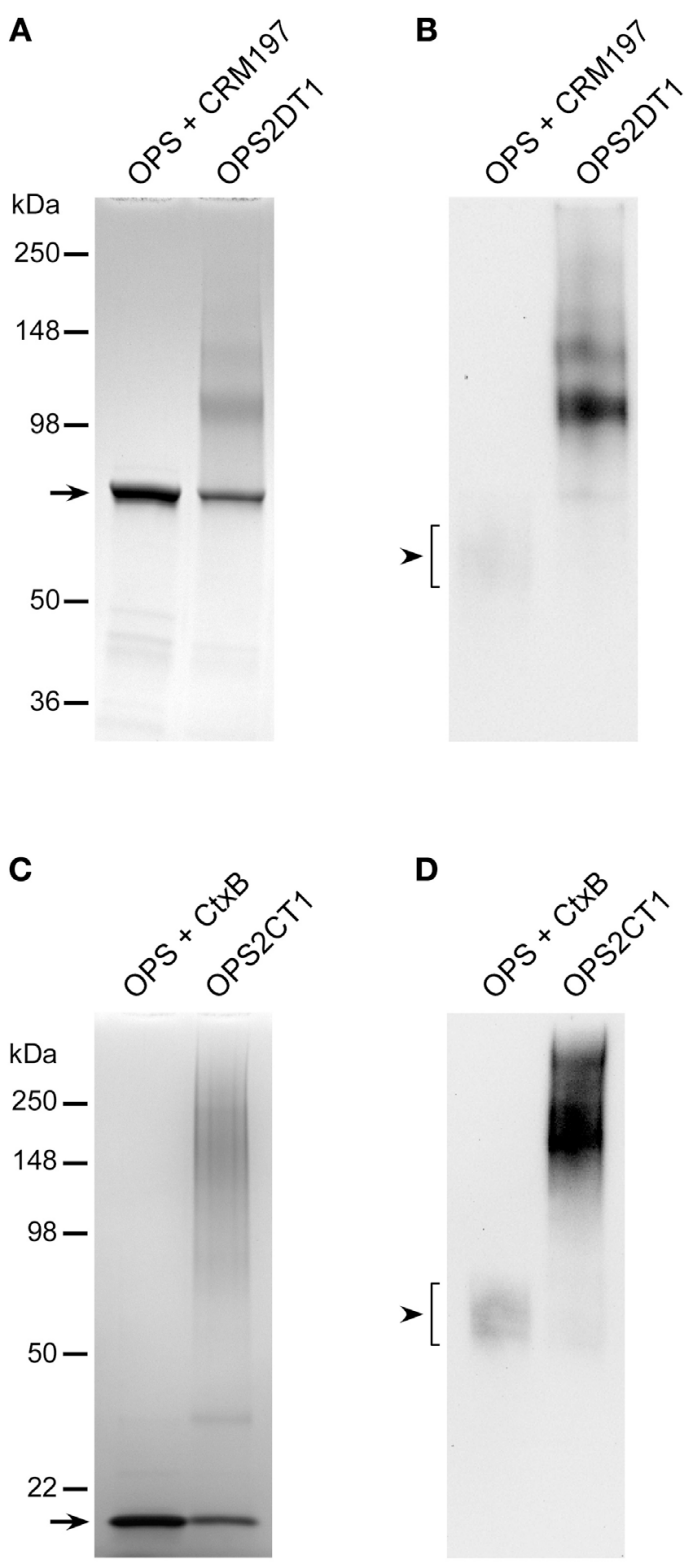
**Physical analysis of *B. thailandensis* OPS-CRM197 and OPS-CtxB glycoconjugates. (A** and **C)** SDS-PAGE and Coomassie Blue staining was used to confirm the covalent linkage of ZT0715 OPS to CRM197 or CtxB. The OPS + CRM197 or CtxB lanes represent unconjugated controls. The black arrows indicate the positions of the CRM197 or CtxB bands. The positions of protein molecular size standards are indicated on the left. **(B** and **D)** Western immunoblotting was also used to assess the antigenicity of the chemically activated/coupled ZT0715 OPS. OPS was detected using the 3D11 mAb. The black arrowheads indicate the positions of the OPS which binds poorly to the membranes when unconjugated.

Glanders is a re-emerging infectious disease for which no licensed vaccines currently exist. Recent reports have demonstrated that mAbs specific for the OPS antigen expressed by *B. mallei* are passively protective in animal models of infection (Jones et al., 2002; Nelson et al., 2004; Zhang et al., 2011; Aucoin et al., 2012). Because of this, research in our laboratory is focused on developing OPS-based vaccines to immunize humans and animals against diseases caused by this bacterial pathogen. In the present study, we have detailed methodologies to facilitate the isolation of highly purified preparations of *B. mallei*-like OPS from *B. thailandensis*. In addition, we have described a simple strategy for covalently linking the carbohydrate antigen to carrier proteins. Using these approaches, we have also demonstrated that high titer IgG responses can be raised against the carbohydrate component of the OPS-based glycoconjugates. Based upon these observations, studies are currently underway to test the protective capacity of our prototype glycoconjugates in animal models of glanders. Due to obvious limitations regarding the use of cBSA as a carrier for vaccine development, efforts are ongoing to explore the use of licensed carriers (e.g., CRM197, CtxB, ExoA etc.) for this purpose (Figure 6). Additionally, studies are being planned to investigate how differing adjuvants might be used to promote more robust Th1-like IgG responses against the carbohydrate component of the glycoconjugates. Collectively, these studies should provide important insights toward the rational design of efficacious glanders vaccine candidates. It is also anticipated that similar methodologies will be amenable to the development of OPS-based glycoconjugates for immunization against melioidosis.

### Conflict of interest statement

The authors declare that the research was conducted in the absence of any commercial or financial relationships that could be construed as a potential conflict of interest.
